# Bioequivalence Comparison of Two Formulations of Fixed-Dose Combination Glimepiride/Metformin (2/500 mg)Tablets in Healthy Volunteers

**Published:** 2014

**Authors:** Sang-hoon Jung, Jung-woo Chae, Byung-jeong Song, Kwang-il Kwona

**Affiliations:** a *College of Pharmacy, Chungnam National University, Korea.*; b *Medical Department, Novo Nordisk, Pharma Korea, Korea.*

**Keywords:** Metformin, Glimepiride, Combination drug, Bioequivalence, Pharmacokinetic properties

## Abstract

Glimepiride/metformin (2/500 mg) is an oral antihyperglycemic agent for the treatment of type 2 diabetes. A generic glimepiride/metformin (2/500 mg) fixed-dose combination (FDC) tablet was developed recently. This study was designed to collect data for submission to Korean regulatory authorities to allow the marketing of the test formulation. We evaluated the comparative bioavailability and tolerability of the test and reference formulations in healthy male adult volunteers. This single-dose, randomized, double-blind, two-way crossover trial was conducted at Bestian Medical Center in Bucheon, Korea. In total, 40 male Korean volunteers were enrolled. The subjects were randomized to receive an FDC tablet containing the glimepiride/metformin (2/500 mg) test or reference formulation, and pharmacokinetic(PK) parameters were measured. After a 1-week washout period, the other formulation was administered and the PK parameters were measured again. The C_max _and AUC_t _were determined from blood samples obtained at 0, 0.5, 1, 1.5, 2, 2.5, 3, 3.5, 4, 5, 6, 8, 12, and 24 h after drug administration. Bioequivalence was considered established if the 90% CIs of the geometric mean ratios(GMRs) of the test-to-reference formulations for C_max _and AUC_t _were within the predetermined regulatory range of 80-125%. In total, 40 healthy male subjects were enrolled and completed the study (mean [SD] age, 23.2[2.26]years[range, 19-30years];weight, 68.95[8.30]Kg[range, 52.0-87.0 Kg]; and height, 175.4[5.34] cm[range, 164-189 cm]). The GMRs(90% CI) of the glimepiride C_max _and AUC_t _were 1.006(0.947-1.069) and 1.010(0.953-1.071), respectively. For metformin, the values were 1.019(0.959-1.083) and 1.035(0.989-1.084), respectively. The test and reference formulations had similar PK parameters. The test formulation of glimepiride/metformin (2/500 mg) FDC tablets met the Korean regulatory criteria for bioequivalence.

## Introduction

Type 2 diabetes mellitus (DM) is a complex disease characterized by a range of metabolic defects, imbalances, and abnormalities affecting multiple organ systems. Three key defects herald the onset of hyperglycemia and type 2 diabetes: (1) impaired insulin action/insulin resistance in muscle, liver, and adipose tissue;(2) elevated prandial and postprandial hepatic glucose production, resulting, in part, from defects in glucagon regulation; and (3) diminished insulin secretion, resulting from defects in β cell function and mass (1-6).Various medications have been approved for the treatment of type 2 D Min many countries. Combination treatments decrease the risk of medication non-compliance and have cost-saving effects (7,8).The most common combination regimens are (I) a sulfonylurea with metformin or a thiazolidinedione (TZD) and (II) metformin with a TZD (9). Metformin with a sulfonylurea causes greater reductions in HbA1c than either drug alone (10-12).

Glimepiride is an oral sulfonylurea antihyperglycemic agent. The primary mechanism of action of glimepiride in lowering blood glucose appears to be dependent on stimulating the release of insulin from functioning pancreatic β cells. Extrapancreatic effects may also play a role (13-15).Glimepiride is indicated for the treatment of type 2 DM at are recommended dosage of 18 mg/day for adults and is typically administered with other antihyperglycemic agents such as metformin or TZDs to provide adequate glycemic control (9,16).

Metformin, a biguanide glucose-lowering agent, is commonly used to manage type 2 DM(16). Monotherapy with metformin is used as an adjunct to diet to manage type 2 DM in patients whose hyperglycemia cannot be controlled by diet alone (17).Metformin may also be used in combination with other antidiabetic agents in patients with type 2 DM who do not achieve adequate glycemic control with a sulfonylurea agent alone (18). The glucose-lowering effect of metformin is primarily the result of reduced hepatic glucose output, through the inhibition of gluconeogenesis and glycogenolysis (19).

The sulfonylurea glimepiride might offer some advantages as a component of a fixed-dose combination(FDC) tablet with metformin due to its more prominent extrapancreatic activity and more favorable safety profile, compared with other sulfonylureas (20-23).The combination of glimepiride and metformin was approved by the US Food and Drug Administration in 1999.

The present bioequivalence study was designed to provide data to submit to regulatory authorities to allow marketing of the test formulation, which is the first generic glimepiride/metformin (2/500 mg) FDC tablet formulation approved in Korea. In this study, we compared the pharmacokinetic (PK) properties, safety profiles, and relative bioavailability of the generic (test) and brand(reference) glimepiride/metformin FDC tablets in healthy male Korean volunteers. 

## Experimental

*Subjects*

In total, 40 healthy Korean male volunteers participated. The subjects ranged in age from 19-30 years (mean SD, 23.2 2.26 years) and weighed between 52 and 87 Kg (mean SD, 68.958.29Kg). The demographic characteristics of the subjects are summarized in Table 1.All laboratory tests were performed at the Department of Laboratory Medicine, Bestian Medical Center(BMC, Bucheon, Korea), which has been accredited by the Korean Association of Quality Assurance for Clinical Laboratories.

**Table 1:** Characteristics of the 40 healthy Korean male subjects

**Characteristic**	**Mean**	**SD**	**Median**	**Range**
Age(years)	23.2	2.3	23.0	19-30
Body weight(Kg)	68.9	8.3	67.5	52-87
Height(cm)	175.4	5.3	174.0	164-189
DOO(%)	100.9	8.9	99.0	80-120
AST(U/L)	22.1	4.7	20.0	17-32
ALT(U/L)	21.9	7.2	20.5	11-41
ALP(U/L)	245.1	45.2	245.5	161-359
Albumin(g/dL)	4.7	0.3	4.8	4.1-5.4
Total bilirubin(mg/ dL)	0.9	0.8	0.9	0.5-1.6
BUN(mg/dL)	12.2	3.4	12.1	5.6-22.3
SCr(mg/dL)	0.9	0.2	0.9	0.5-1.3
CLcr(mL/min)	1.2	0.3	1.1	0.8-2.1

DOO Density of obesity, AST Aspartate aminotransferase, ALT Alanine aminotransferase, ALP Alkaline phosphatase, BUN Blood urea nitrogen, SCr Serum creatinine, CLcr Creatinine clearance

No subject had any significant cardiac, hepatic, renal, pulmonary, neurological, gastrointestinal, or hematological disorder, as determined by medical history and physical examinations; the latter included assessments of the subjects’ vital signs and clinical laboratory values(hematology, blood chemistry, and urinalysis). Furthermore, each subject was physically normal and had no previous history of significant illness or hypersensitivity to any drug. Finally, the subjects were asked to refrain from taking alcohol, caffeine, and any other drugs for at least 1week before and throughout the study period.

The study protocol was approved by the Institutional Review Board of BMC. All procedures were performed according to Good Clinical Practice guidelines and the Declaration of Helsinki for biomedical research (24) involving human subjects. All participants provided written informed consent to join the study before the screening test for eligibility.

*Study design and materials*

This bioequivalence study was conducted as a randomized, single-dose, two-period, two-sequence, crossover study. There were two treatment periods separated by a 1week washout period, which was more than five times the half-life, as determined in previous studies (25,26). A table of random numbers was used to assign subjects in a 1:1 ratio to receive the test drug or reference drug. All subjects fasted for at least 10h before administration. The study drug was given with 240 mL of water, and all subjects abstained from food until 4 h after dosing. After a washout period of 7 days, the same procedure was repeated with the other formulation. Dietary, smoking, and drug-herbal product restrictions were maintained throughout the study period. 

*Blood sampling*

Blood samples for PK analyses were collected before and at 0.5, 1, 1.5, 2, 2.5, 3, 3.5, 4, 5, 6, 8, 12, and 24 h after drug administration. They were drawn through an indwelling intravenous catheter placed in the forearm vein and transferred to a heparinized tube. After the first 1 mL of blood was discarded, a 9 mL sample was collected. Next, the cannula was flushed with 1 mL of normal saline to prevent clotting. Following centrifugation (3,000*x g*, 10min, 4 °C), plasma samples were transferred to microcentrifuge tubes and immediately stored at -70 °C until analysis. After a washout period of 7 days, the study was repeated in the same manner to complete the crossover design.

*Measurement of plasma glimepiride and metformin*

**Figure 1:**
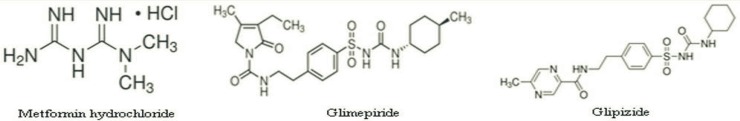
Structural representation of metformin, glimepiride and glipizide

All plasma samples were handled and analyzed by BIOSUNTEK Laboratory Co., *Ltd.*(Sungnam, Korea) using validated bio analytical methods (27-29). Plasma concentrations of glimepiride and metformin were determined using validated high-performance liquid chromatography (HPLC,1200 series; Agilent Technologies, Inc., Santa Clara, CA, USA) coupled with Triple Quad LC/MS/MS(Agilent Technologies, Inc.). A Cap cell PAK C_18_UG80 (3.0 × 35 mm, 5 µm) column was obtained from Shiseido Co., *Ltd.*(Tokyo, Japan). Briefly, 200 µL of plasma was spiked with the internal standard (glipizide). Liquid-liquid extraction was done using 0.2% formic acid in acetonitrile for glimepiride and metformin. The mobile phase consisted of a mixture of methanol and 20 mM ammonium formate (80:20, v/v). The flow rate of the mobile phase was 0.4 mL/min and the injection volume was 10 µL. Glimepiride, metformin, and glipizide (Sigma-Aldrich, St. Louis, MO, USA) were detected by MS with an electrospray ionization interface, operating under positive selected reaction monitoring MS/MS conditions at the following mass transitions: 491.3→352 *m/z* for glimepiride, 130.1→71.1 *m/z* for metformin, and 446.2→321.2 *m/z* for glipizide. Figure 1 shows structural representations of metformin, glimepiride, and glipizide.

For the quantification of glimepiride, the method had a linear quantifiable range of 2400 ng/mL and a γ^2^ of 0.997. The lower limit of quantitation(LLOQ) was 2 ng/mL. The intraday precision ranged from 3.31-8.27%, while the inter day precision ranged from 0.41-5.55%. The intraday accuracy was 102.6-103.6% while the inter day accuracy was 102.0-103.6%. The analyte was stable in human plasma following three freeze-thaw cycles, in plasma after storage for 6 h at room temperature, in stock solution after storage for 6 h at room temperature, and in the HPLC auto sampler after storage for 24 h.

For the quantification of metformin, the method had a linear quantifiable range of 5-2000 ng/mL and a γ^2^ of 0.998. The LLOQ was 5 ng/mL. The intraday precision ranged from 0.96-2.18%, while the inter day precision ranged from 3.26-9.77%. The intraday accuracy was 88.9-96.7% while the inter day accuracy was 98.2-100.2%. The analyte was stable in human plasma following three freeze-thaw cycles, in plasma after storage for 6 h at room temperature, in stock solution after storage for 6 h at room temperature, and in the HPLC auto sampler after storage for 24 h.

*PK analysis*

Changes in the plasma concentrations of glimepiride and metformin in each subject were analyzed using non-compartmental PK models and individual PK parameters were assessed using the BA-CALC program(KFDA, 2008, 1.0.0, Korea).PK parameters included the area under the plasma concentration-versus-time curve from 0 h to the last measurable concentration (AUC_t_), which was calculated using the linear trapezoidal rule, maximum plasma concentration (C_max_),and time required to reach the maximum plasma concentration (T_max_).The terminal elimination rate constant(λ_Z_) was estimated using linear regression of the log-linear decline of individual plasma concentration-time data. The individual t_½_was calculated for each subject as ln2/λ_Z_. AUC_∞_ was calculated as AUC_t_+ C_last_/λ_Z_, where C_last_ was the last measurable concentration (30).

*Statistical analyses*

The demographic characteristics of the enrolled subjects were summarized using descriptive statistics. We used a sample size of 40, as required by the Korean Food and Drug Administration, because of the potential for dropouts during the study. Statistical analysis was performed using the K-BE test program(KFDA, 2007, 1.1.0, Korea).The GMRs of the test and reference values for AUC_t_ and C_max_ were calculated. The formulations were assumed bioequivalent if the 90% CIs for the GMRs of the test formulation to the reference formulation were from 0.8 to 1.25.

## Results and Discussion

In total, 40 adult Korean male volunteers participated in this bioequivalence study. Physical examinations conducted before starting the study indicated that the subjects were healthy. There were no dropouts during the study period. No clinical AEs were reported.

**Figure 2:**
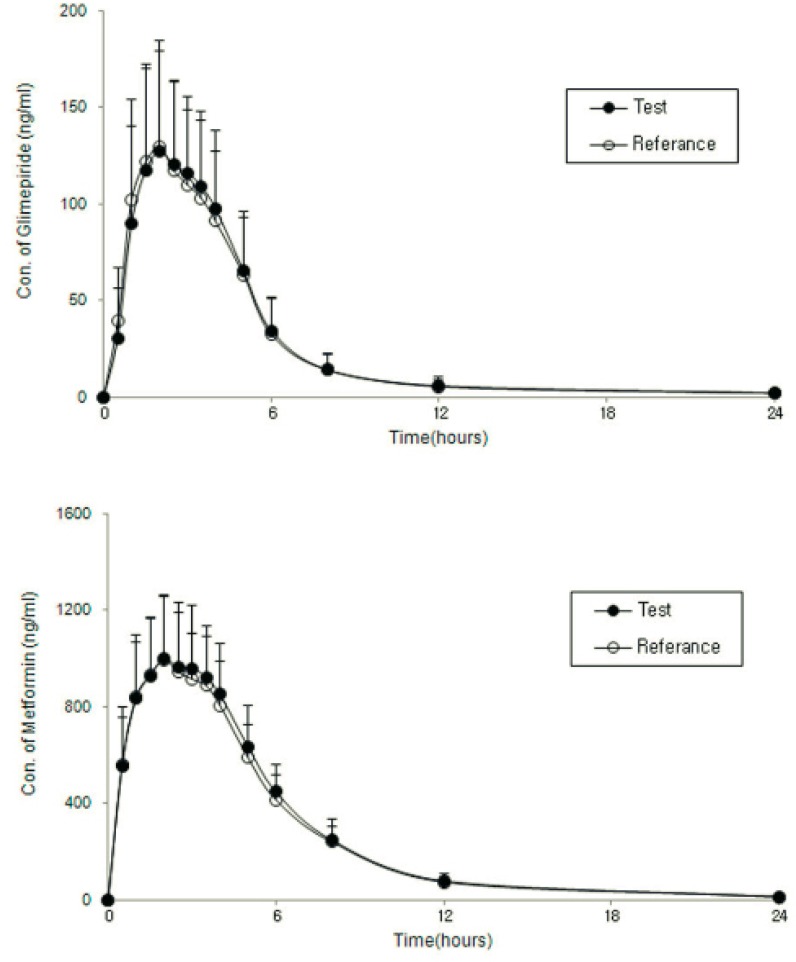
Mean(SD) plasma concentration-time profiles of glimepiride and metformin after administration 2 formulations of a glimepiride/metformin 2/500 mg FDC tablet

Figure 2 shows the mean ± SD plasma concentration-time profiles of glimepiride and metformin after administration of two formulations of a glimepiride/metformin (2/500 mg) FDC tablet, and Table 2 shows the values of the PK parameters for the test and reference formulations. The formulations had very similar AUC_t_ and C_max_ values. Table 3 shows the GMRs of the reference and test drugs. The GMRs (90% CIs) of C_max_ and AUC_t _for glimepiride and metformin ranged from 0.8 to 1.25. This meets the Korean regulatory criteria for bioequivalence.

**Table 2:** Pharmacokinetic parameters following administration of the test and reference formulations of glimepiride/metformin 2/500 mg FDC tablet, in fasting, healthy male subjects(n=40)

(**A) Non-compartmental Analysis of Glimepiride**		
**Pharmacokinetic Results**	**Test**	**Reference**
	Mean(SD)(CV%)	Mean(SD)(CV%)
AUC_t_, ng∙h/mL	634.0(253.5)(40.0)	625.0(253.9)(40.6)
C_max_, ng/mL	144.0(49.8)(34.6)	143.3(51.3)(35.8)
t_½_, h	3.0(2.0)(68.5)	2.9(1.9)(65.7)
T_max_, h†	2.2(0.8,4)	2.0(0.9,5.0)
**(B) ** **Non-compartmental** ** Analysis of Metformin**		
**Pharmacokinetic Results**	**Test**	**Reference**
	Mean(SD)(CV%)	Mean(SD)(CV%)
AUC_t_, ng/h/mL	6391.9(1605.5)(25.1)	6162.2(1433.3)(23.3)
C_max_, ng/mL	1079.1(283.5)(26.3)	1062.4(274.9)(25.9)
t_½_, h	3.3(0.72)(21.6)	3.3(0.72)(21.7)
T_max_, h†	2.2(0.5, 4)	2.5(0.8, 4)

† Median(minimum, maximum).

**Table 3:** Bioavailability of glimepiride and metformin after administration of test and reference drug of glimepiride/metformin 2/500 mg FDC tablet in healthy Korean volunteers

	**Glimepiride**		**Metformin**	
**Parameter**	**Reference(n=20)**	**Test(n=20)**	**Reference(n=20)**	**Test(n=20)**
**C**_max_				
GM*(ng∙h/mL)	577.97	584.13	6105.93	6321.23
GMR^†^(90%CI)	1.01(0.96-1.07)		1.04(0.99-1.08)	
**AUC**_∞_				
GM^†^(ng∙h/mL)	138.86	139.73	1054.26	1074.53
GMR*(90%CI)	1.01(0.95-1.07)		1.02(0.96-1.08)	

* Geometric mean

† Geometric mean ratio

The results of the current study indicate that a generic formulation of glimepiride/metformin (2/500 mg)FDC tablet met the regulatory requirements for assuming bioequivalence to the reference formulation, as established by the Korean Food and Drug Administration.
